# A Smartphone-Based Non-Destructive Multimodal Deep Learning Approach Using pH-Sensitive Pitaya Peel Films for Real-Time Fish Freshness Detection

**DOI:** 10.3390/foods14101805

**Published:** 2025-05-19

**Authors:** Yixuan Pan, Yujie Wang, Yuzhe Zhou, Jiacheng Zhou, Manxi Chen, Dongling Liu, Feier Li, Can Liu, Mingwan Zeng, Dongjing Jiang, Xiangyang Yuan, Hejun Wu

**Affiliations:** 1 College of Science, Sichuan Agricultural University, No.46, Xin Kang Road, Ya’an 625014, China; 2 College of Information Engineering, Sichuan Agricultural University, No.46, Xin Kang Road, Ya’an 625014, China; 3 College of Management, Sichuan Agricultural University, No.211, Hui Min Road, Chengdu 611130, China; 4 College of Food Science, Sichuan Agricultural University, No.46, Xin Kang Road, Ya’an 625014, China

**Keywords:** fish freshness detection, food safety, pH-sensitive film, multimodal deep learning, smartphone-based sensing, real-time monitoring

## Abstract

The detection of fish freshness is crucial for ensuring food safety. This study addresses the limitations of traditional detection methods, which rely on laboratory equipment and complex procedures, by proposing a smartphone-based detection method, termed FreshFusionNet, that utilizes a pitaya peel pH intelligent indicator film in conjunction with multimodal deep learning. The pitaya peel indicator film, prepared using high-pressure homogenization technology, demonstrates a significant color change from dark red to yellow in response to the volatile alkaline substances released during fish spoilage. To construct a multimodal dataset, 3600 images of the indicator film were captured using a smartphone under various conditions (natural light and indoor light) and from multiple angles (0° to 120°), while simultaneously recording pH values, total volatile basic nitrogen (TVB-N), and total viable count (TVC) data. Based on the lightweight MobileNetV2 network, a Multi-scale Dilated Fusion Attention module (MDFA) was designed to enhance the robustness of color feature extraction. A Temporal Convolutional Network (TCN) was then used to model dynamic patterns in chemical indicators across spoilage stages, combined with a Context-Aware Gated Fusion (CAG-Fusion) mechanism to adaptively integrate image and chemical temporal features. Experimental results indicate that the overall classification accuracy of FreshFusionNet reaches 99.61%, with a single inference time of only 142 ± 40 milliseconds (tested on Xiaomi 14). This method eliminates the need for professional equipment and enables real-time, non-destructive detection of fish spoilage through smartphones, providing consumers and the food supply chain with a low-cost, portable quality-monitoring tool, thereby promoting the intelligent and universal development of food safety detection technology.

## 1. Introduction

Food safety and quality are core issues that impact public health and the sustainable development of the food supply chain. According to the Food and Agriculture Organization (FAO) of the United Nations, a significant amount of food is wasted globally each year due to spoilage, resulting in economic losses totaling hundreds of billions of dollars. Among these, aquatic products, due to their perishable nature, are one of the categories most severely affected by waste [1]. With the continuous growth in global consumption of fresh food, particularly high-protein aquatic products such as fish, which are widely circulated worldwide (for instance, fish consumption in the Asia–Pacific region maintains a high annual growth rate, significantly outpacing other animal-derived foods), ensuring the freshness of food during transportation, storage, and sales has become an urgent challenge. Taking salmon as an example, foodborne illnesses caused by its spoilage account for a notable proportion of global seafood-related cases, underscoring the urgent need for efficient detection technologies.

Fish meat, rich in protein and moisture, is highly susceptible to spoilage due to microbial action and endogenous enzyme catalysis, resulting in the production of volatile basic substances (such as ammonia and trimethylamine) and biogenic amine compounds. This leads to an increase in total volatile basic nitrogen (TVB-N) content, a surge in total microbial counts, and significant changes in pH values [2]. The deterioration of these biochemical indicators not only affects the sensory quality of fish meat but also poses a risk of foodborne illnesses, threatening consumer health. Research by the World Health Organization (WHO) indicates that histamine produced by fish spoilage can lead to allergic poisoning, which can be life-threatening in severe cases [3].

Traditional methods for assessing fish freshness primarily rely on laboratory-based chemical analyses, such as the colorimetric determination of total volatile basic nitrogen (TVB-N) and plate count methods for total viable count (TVC). Although these techniques offer reliable results, they are typically complex, time-consuming, and costly, requiring specialized equipment and trained personnel, which limits their applicability for real-time monitoring or large-scale use in consumer-oriented scenarios [4].

To improve detection efficiency, recent studies have explored rapid sensing technologies based on instruments like electronic noses and near-infrared (NIR) spectrometers. For example, Yu et al. (2020) employed NIR spectroscopy combined with chemometric models to perform non-destructive freshness assessment of tilapia fillets [5]. However, such instruments are often expensive, bulky, and energy-intensive, making them difficult to deploy widely in cold chain logistics or retail environments.

With the significant advancements in image processing and edge computing capabilities of smartphones, visual recognition-based food detection methods have gained increasing attention. Zhang et al. (2021) proposed a TVB-N detection strategy that integrates smartphone-based image acquisition with a nanocomposite-sensitive film, enabling visual monitoring of fish spoilage levels [6]. This approach offers advantages such as low cost and portability, making it suitable for preliminary evaluations in household or retail contexts. Nevertheless, due to its reliance on static image input, the system is highly susceptible to environmental interferences such as lighting and viewing angle, and fails to capture the temporal dynamics of chemical indicators, thereby limiting its effectiveness in detecting early-stage spoilage. While visual monitoring using indicator films, as in the aforementioned study, can provide an intuitive sign of spoilage, relying solely on naked-eye observation presents inherent limitations. Human color perception is subjective and can vary significantly between individuals and under different ambient lighting conditions, leading to inconsistent assessments. Furthermore, such manual visual checks often lack the capability for quantitative analysis, objective record-keeping essential for quality control and traceability, or the implementation of standardized assessment protocols across diverse users and environments. To address these shortcomings and harness the advanced functionalities of ubiquitous mobile technology, the integration of smartphone-based sensing with indicator films offers substantial added value. Smartphone-based systems can provide several distinct advantages:

(1) **Objective Quantification:** Smartphone cameras, when coupled with sophisticated image-processing algorithms and deep learning models as proposed in this work, can objectively capture and quantify subtle color changes that might be indicative of early-stage spoilage, often beyond the consistent discernment capabilities of the human eye. This leads to more precise and reproducible freshness assessments.
(2) **Data Logging and Traceability:** As demonstrated by the application developed in this study (Section 4.6), smartphones can automatically record detection results, including timestamps, images, and associated chemical data, thereby creating a valuable digital log for quality assurance, supply chain management, and traceability.
(3) **Standardization:** A smartphone-based detection system can enforce a standardized protocol for image acquisition and analysis, minimizing variability introduced by different operators or viewing conditions.
(4) **Enhanced Accessibility and Portability:** The widespread availability, low cost, and inherent portability of smartphones make them ideal tools for convenient on-site and real-time freshness monitoring by a broad range of users, from consumers to food industry professionals.
(5) **Connectivity and Advanced Analytics Potential:** Smartphone-derived data can be easily shared or integrated with cloud platforms for remote monitoring, trend analysis, or integration into larger food safety management systems.

Therefore, the synergy between pH-sensitive films and smartphone-based intelligent sensing provides a robust, objective, and scalable paradigm for modern food quality monitoring.

In recent years, natural pigment-based pH-sensing films have emerged as promising alternatives for intelligent food monitoring. Our group previously developed a red cabbage puree-based film, incorporating polyvinyl alcohol to enhance mechanical properties and water resistance. This film exhibited strong color sensitivity to pH variations and effectively indicated fish spoilage through visible chromatic transition [7]. In a subsequent study, we utilized red pitaya peel as both a film-forming matrix and a source of betacyanins, and applied high-pressure homogenization to improve film uniformity and sensitivity. The resulting pH-responsive films showed enhanced tensile strength and high ammonia sensitivity, enabling direct visual monitoring of fish freshness under ambient conditions [8]. These two studies laid the foundation for integrating intelligent indicator materials with digital recognition systems.

To enhance the modeling of spoilage dynamics, multimodal learning approaches have received growing interest. Lee et al. (2019) introduced the MildInt framework [9], which incorporated long-term temporal features for biological signal modeling and emphasized the integration of visual and non-visual modalities. However, this method was primarily designed for genomic and phenotypic data fusion and did not address the low robustness of visual modalities under dynamic monitoring. In the context of food detection, Cao et al. (2024) developed an intelligent hydrogel system based on UCNPs@SiO_2_-phenol red nanoprobes, capable of real-time pH sensing with fluorescence output [10]. Despite its sensing accuracy, the system depends on costly materials and complex fabrication processes, hindering its large-scale consumer application. Furthermore, most existing multimodal fusion methods rely on static weighting or simple feature concatenation, lacking the ability to dynamically adjust the relative importance of different modalities across spoilage stages, and failing to resolve the temporal misalignment between biochemical changes and visual cues.

Based on the above analysis, this study proposes an integrated approach for fish freshness detection that combines a natural pH-sensitive indicator film with multimodal deep learning. A multimodal dataset was constructed by synchronously collecting smartphone images and chemical indicators (pH, TVB-N, and TVC). A lightweight Multi-scale Dilated Fusion Attention (MDFA) module was introduced to enhance visual feature extraction, followed by a Temporal Convolutional Network (TCN) to model dynamic patterns in chemical indicators across spoilage stages. Finally, a Context-Aware Gated Fusion (CAG-Fusion) mechanism was designed to adaptively integrate image and chemical features [11,12]. The resulting model, named FreshFusionNet, enables real-time, visual detection of fish spoilage on smartphones. This work provides a practical and scalable solution for low-cost, high-performance aquatic product quality monitoring.

## 2. Materials and Data Preprocessing

### 2.1. Preparation of Pitaya Peel pH Indicator Films

**Figure 1:**
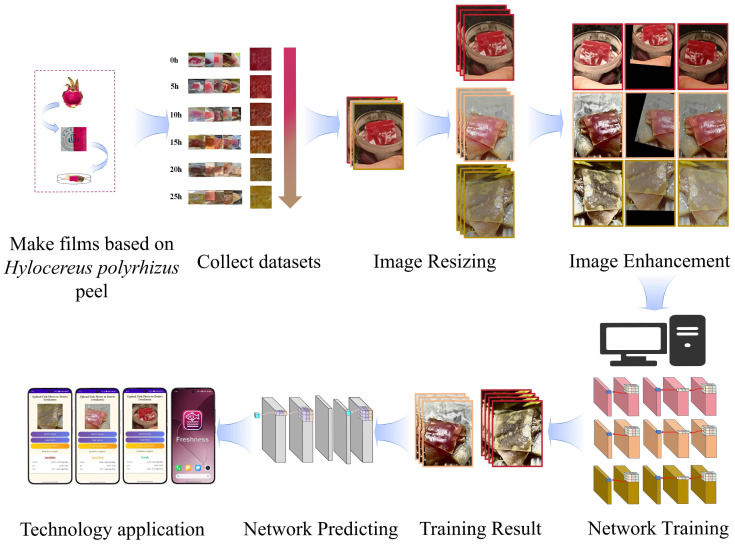
Process diagram of freshness detection using pH-sensitive pitaya peel films and multimodal deep learning.

This study prepared pitaya peel pH indicator films using high-pressure homogenization technology and established an experimental system for fish freshness detection based on multimodal data collected via smartphones [13], combined with data preprocessing and enhancement strategies (Figure 1). The experimental materials utilized red-skinned pitaya (Hylocereus polyrhizus) sourced from Sichuan Province, China, with a peel thickness ranging from 2.5 to 3.0 mm. After washing with deionized water to remove residual pulp, the peels were cut into 5 × 5 mm pieces and placed in a ventilated drying oven at 45 °C for 24 h until the moisture content was reduced to below 5%. The dried fruit peel fragments were then mixed with deionized water at a 1:10 mass-to-volume ratio. This mixture was homogenized for 10 cycles at a pressure of 60 MPa using a high-pressure homogenizer (APV-1000, APV, an SPX Flow Brand, Silkeborg, Denmark), a process known to effectively reduce particle size and improve dispersion for creating uniform films. This HPH process reduced the particle size to the 50–100 nm range, forming a uniformly dispersed film-forming dispersion (FFD) [14]. The FFD was subsequently poured into a polytetrafluoroethylene mold (10 × 10 cm), transferred to a vacuum drying oven (DZF-6050, Shanghai Jinghong Laboratory Instrument Co., Ltd., Shanghai, China; temperature control accuracy ±1 °C), and dried at 45 °C under a vacuum of −0.095 MPa for 8 h. This procedure yielded indicator films with a thickness of approximately 0.09 mm and high transparency. The fundamental pH-responsive nature of the prepared films, exhibiting characteristic color changes from reddish to yellowish hues upon transitioning from acidic/neutral to alkaline environments, was confirmed through preliminary evaluations using standard laboratory techniques including colorimetry [15]. These initial assessments established the film’s capability to visually indicate shifts towards alkalinity [16]. Furthermore, the films possessed adequate mechanical integrity for practical handling and application, and demonstrated good stability suitable for typical cold chain transportation scenarios [17]. A detailed quantitative characterization of the film’s sensing performance, primarily referencing our established findings in [8], is provided in Section 2.2.

### 2.2. Performance Characterization of the HPH-Treated Indicator Film

The suitability of the HPH-treated RPP indicator film (prepared under optimal conditions: 60 MPa, 10 cycles) as a reliable sensor for fish freshness monitoring has been previously established and characterized in detail [8]. Key performance aspects from this published work, substantiating its practical utility in the current study, are summarized below:

**pH Sensitivity:** The aforementioned study [8] demonstrated that the film-forming dispersions (FFDs) of HPH-treated RPP exhibit distinct color changes in response to a wide pH range (pH 2–12), transitioning from red in acidic/neutral conditions (pH 3–7) to purplish and eventually yellow under strong alkaline conditions (pH 12) (Figure 3e therein). UV-Vis spectroscopy confirmed changes in the betalain absorption spectra corresponding to these pH variations (Figure 3f therein). While a detailed quantitative table of ΔE values for the solid film across multiple pH points is not explicitly presented in this specific publication, the observed significant color transitions of the FFDs, which form the basis of the film, confirm its fundamental pH-responsive nature and sensitivity to alkaline shifts indicative of spoilage.

**Ammonia Sensitivity and Response Time:** The indicator film’s high sensitivity and rapid response to volatile ammonia (3 M), a key indicator of fish spoilage, were quantitatively evaluated [8]. Data (Table 5 therein) showed that a significant color change (ΔE>12, indicating a clear visual response) occurred within approximately 40 s of exposure. The response curve derived from these data (ΔE vs. time) indicated that the total color difference reached a substantial value of ΔE≈56 after 8 min and ΔE≈65 after 10 min, at which point the color change had largely saturated, indicating the “time to full color change” or stabilization. This demonstrates a fast and strong response suitable for real-time monitoring. The rapid and significant ΔE change confirms its sensitivity to ammonia and its timely responsiveness.

**Irreversibility:** Consistent with the known chemical degradation of betalains under alkaline conditions, as discussed in relation to the pH response [8], the color change induced by high pH or ammonia is considered irreversible. This characteristic is advantageous for monitoring the cumulative and non-reversible process of food deterioration.

**Storage Stability:** The film’s stability under various storage conditions was also reported [8]. Stability tests (Figure 8 therein) showed minimal color change (ΔE typically < 5 or <12, depending on interpretation of “minimal” from the figure) for at least 14 days under refrigerated (4 °C) and frozen (−18 °C) storage. While degradation was faster at ambient temperature (25 °C), the film maintained sufficient stability for the typical duration of short-term spoilage monitoring relevant to the current study.

These previously established performance characteristics, including its qualitative pH sensitivity, quantified high sensitivity and defined response times to ammonia (supported by ΔE data and response curve information from Table 5 in [8]), and confirmed irreversibility and adequate storage stability, confirming that the HPH-treated RPP indicator film employed in this study is a suitable sensing material for the visual and smartphone-based detection of fish freshness, and thus substantiating its practical utility.

### 2.3. Experimental Setup for Freshness Detection

**Figure 2:**
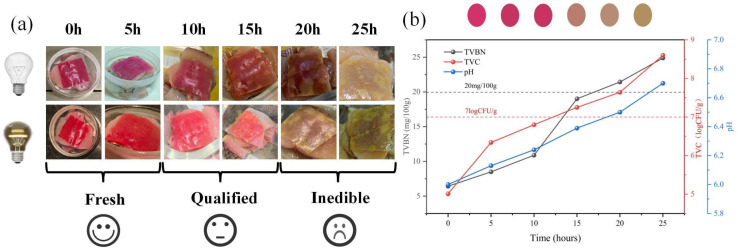
(**a**) Visual changes of pH-sensitive pitaya peel films during fish spoilage at different time points. (**b**) Dynamic changes of chemical indicators (TVB-N, TVC, pH) during fish spoilage.

The experimental samples were selected from fresh sea bass (Lateolabrax japonicus), each weighing 500 ± 50 g, and were cut into 50 g pieces before being placed in a 25 °C incubator to simulate the natural spoilage process, with continuous monitoring over 25 h. Data collection was conducted using a Xiaomi Civi4 Pro smartphone, Xiaomi Corporation, Beijing, China (main camera: 50 megapixels, 1/1.55-inch sensor, f/1.63 aperture) for photography. To simulate the complexity of real-world application scenarios, three lighting conditions were established: natural light (D65 standard light source, illumination intensity of 1000 lux), indoor cool light (LED 5000 K, 500 lux), and indoor warm light (LED 3000 K, 300 lux). Five shooting angles were selected: 0° (front view), 30°, 60°, 90° (top view), and 120° (side view), covering common inspection perspectives. Starting from the onset of spoilage (t = 0), images were captured every 5 h, totaling six time points. At each time point, 10 images were taken under different combinations of lighting and angles, resulting in a total of 3600 raw images (Figure 2a). The synchronously recorded chemical indicators included pH value, total volatile basic nitrogen (TVB-N), and total viable count (TVC) (Figure 2b) [18]. The determination of TVB-N was conducted in accordance with the Chinese National Standard [19].In this process, 1 g of fish sample was added to 10 mL of 7.5% trichloroacetic acid solution, homogenized, and then centrifuged at 4000 rpm for 10 min. The supernatant was reacted with Nessler’s reagent, and the absorbance was measured at 532 nm using a Shimadzu UV-2600 spectrophotometer, Shimadzu Corporation, Kyoto, Japan. The TVB-N concentration (mg/100 g) was calculated using a standard curve [20]. The determination of TVC followed the standard [21], where the fish homogenate was serially diluted and spread on Plate Count Agar (PCA) medium, incubated at 37 °C for 48 h, and the colony-forming units (CFU/g) were counted. The pH value was obtained by extracting the RGB mean value from the central area (100 × 100 pixels) of the indicator membrane using ImageJ software (v1.53), which was then converted to the actual pH value based on a pre-calibrated curve (cubic polynomial fitting, R^2^ = 0.98), thereby minimizing the risk of contamination caused by direct contact with the fish sample.

**Figure 3:**
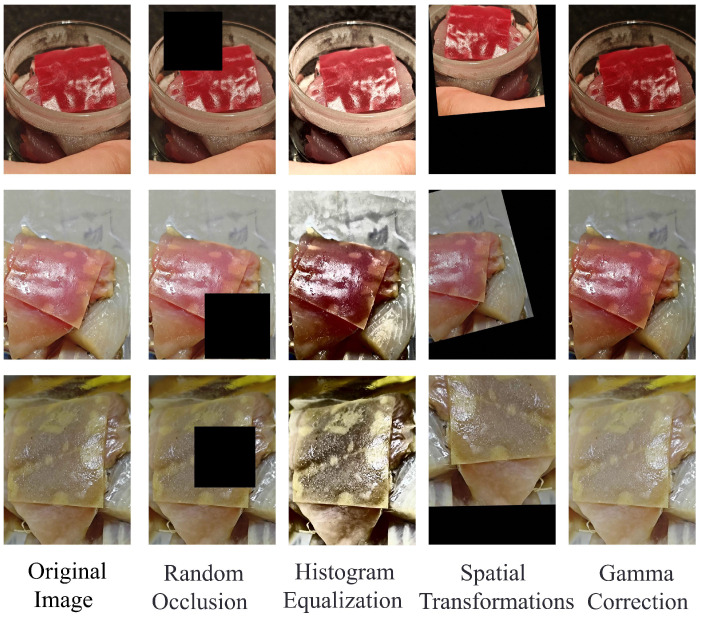
Data augmentation techniques for fish freshness detection images.

To enhance the model’s adaptability to complex environments, all original images are uniformly resized to 512 × 512 pixels using a bilinear interpolation algorithm [22], thereby meeting the input size requirements of the MobileNetV2 network. To address variations in lighting conditions, color normalization techniques are applied to linearly map the RGB channel values from 0–255 to the [0,1] range, which reduces the interference of light intensity on color features. In the data augmentation process, global contrast enhancement is first achieved through histogram equalization, combined with gamma correction (γ=0.5–1.5) to dynamically adjust the brightness distribution of the image [23]. This effectively strengthens the dynamic response range of the indicator film’s color characteristics. The spatial transformation module employs a combination strategy of random rotation ($\pm 15^{\circ }$), horizontal flipping (50% probability), and random scaling (0.8–1.2 times) to simulate the scale and orientation differences caused by shooting angle deviations and variations in object distance during practical applications. A specially designed random occlusion mechanism generates mask regions that cover 10–20% of the image area at random positions, effectively enhancing the model’s robustness to complex scenarios such as lens foreign object occlusion and local reflection of packaging film. Following the aforementioned preprocessing and augmentation operations (Figure 3), the dataset was expanded from 3600 to 7200 images and divided into training and validation sets at an 8:2 ratio. This ensures a balanced distribution of samples across all stages of spoilage (fresh, qualified, inedible), thereby preventing classification bias in the model due to data skewness.

## 3. Methods

### 3.1. Overall Model Architecture Design

**Figure 4:**
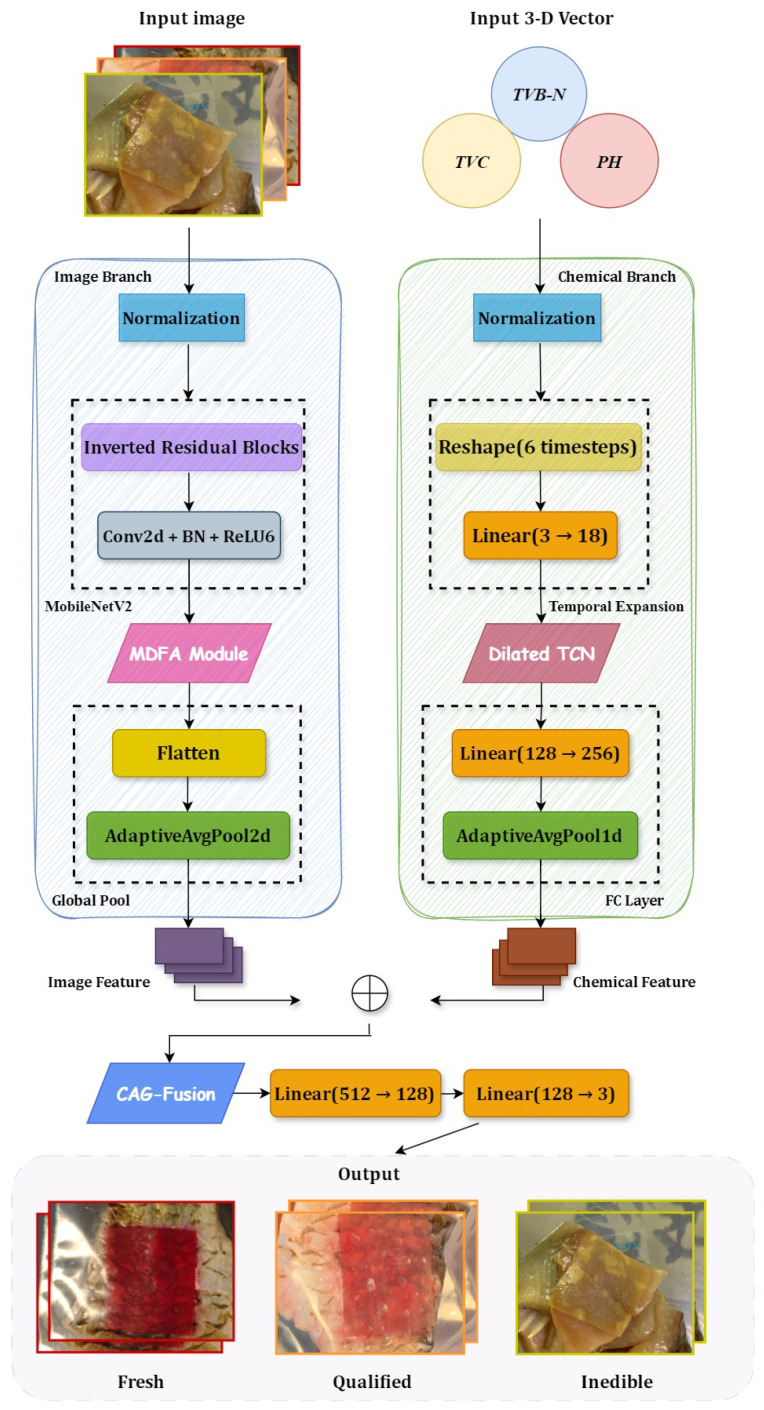
Architecture of the FreshFusionNet model for fish spoilage assessment.

The multimodal freshness detection model proposed in this paper, termed FreshFusionNet, is designed for smartphone-based, real-time detection of fish spoilage. It achieves this by deeply integrating visual information from a pH-sensitive indicator film with quantitative chemical data. As illustrated in Figure 4, FreshFusionNet’s overall architecture comprises three main components:

(1) An image feature extraction branch, responsible for processing visual cues from the pH indicator film.
(2) A chemical data-processing branch, specifically designed to model the temporal dynamics of sequentially collected chemical indicators (pH, TVB-N, TVC) using a Temporal Convolutional Network (TCN).
(3) A Context-Aware Gated Fusion (CAG-Fusion) module, tasked with adaptively integrating the features from these distinct modalities.

These modules operate synergistically. This collaborative approach aims to fully leverage the intuitiveness of image data alongside the quantitative advantages of chemical data, ultimately producing robust classification results for fish freshness.

The image feature extraction branch begins with MobileNetV2 as its backbone network. This backbone has been optimized to meet the detection requirements for color changes in the indicator membranes. Subsequently, a Multi-scale Dilated Fusion Attention (MDFA) module is integrated at the end of the MobileNetV2 feature extractor. The MDFA module employs parallel dilated convolutions, specifically with dilation rates of 6 and 12. This configuration enables it to capture both local details and global trends in the color of the indicator membrane. The final output of this image branch is a 256-dimensional feature vector, which characterizes the observed color change patterns. Furthermore, the channel attention mechanism within MDFA dynamically adjusts the weights of different color channels. This adjustment helps to reduce the impact of lighting variations on the detection results.

The chemical data-processing branch is tailored to handle time-series data from quantitative indicators, including pH, total volatile basic nitrogen (TVB-N), and total viable count (TVC). As further elaborated in Section 3.3, this branch first transforms the discrete chemical measurements (pH, TVB-N, TVC) into pseudo time-series sequences. This step simulates the dynamic changes that occur during the spoilage process. Following this, a Temporal Convolutional Network (TCN) is employed. The TCN incorporates 1D convolutional kernels with dilation rates of 2 and 5, allowing it to extract both short-term fluctuations and long-term trends from the chemical indicators [24]. The resulting chemical features are then mapped to a 256-dimensional space using a fully connected layer. This design significantly enhances the model’s sensitivity to subtle changes, particularly in the early stages of spoilage.

To effectively integrate these image and chemical features, a Context-Aware Gated Fusion (CAG-Fusion) mechanism is proposed [25]. This mechanism begins by encoding the image features and chemical features through a lightweight multi-layer perceptron (MLP). The MLP generates context vectors that encapsulate semantic information from each modality [26]. Next, based on a similarity measure between these two context vectors, gating weight values are dynamically calculated. These weights adaptively adjust the fusion ratio of the image and chemical features. This dynamic adjustment is crucial for accurate spoilage assessment. For instance, during the initial stages of spoilage, the pH value of fish changes significantly, while the color change of the indicator film might be minimal. In such scenarios, the gating mechanism assigns a higher weight to chemical characteristics, enabling the model to capture early fluctuations in TVB-N and TVC. Conversely, in the later stages of spoilage, the color of the indicator film changes noticeably (e.g., from purple-red to yellow), but the chemical indicators may tend to saturate. At this stage, the model adaptively shifts its reliance primarily towards image features for classification. Experimental results demonstrate that this dynamic weight allocation strategy aligns closely with the physical progression of spoilage, evidenced by a correlation coefficient of 0.84 between the gating parameters and pH values.

The fused features are subsequently mapped to freshness classification probabilities via a fully connected network, yielding three prediction outcomes: fresh, qualified, and inedible. To ensure suitability for deployment on mobile devices, FreshFusionNet incorporates a multi-stage lightweight design strategy. First, redundant structures at the end of the MobileNetV2 network are pruned. This pruning reduces the model’s parameter count from 42.56 M to 39.32 M. Second, mixed-precision computation (FP16+INT8) is applied to the attention module. This balances feature extraction accuracy with computational efficiency [27]. Finally, the TensorRT engine is utilized to optimize memory usage and operator execution efficiency [28].

The detailed architectural parameters of the main components and custom modules within the FreshFusionNet model, including the image feature extraction branch, the chemical data-processing branch, and the CAG-Fusion module, are summarized in Table 1. This table provides an overview of the layer configurations, dimensionalities, and key hyperparameters that define the model’s structure and depth. These components will be further elaborated in the subsequent sections.

### 3.2. Multi-Scale Attention Enhancement Module

**Figure 5:**
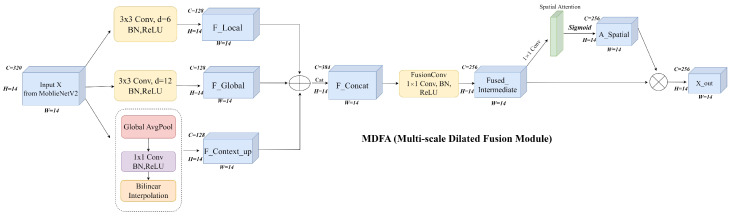
Architecture of the Multi-scale Dilated Fusion Attention (MDFA) module, illustrating the fusion of local, global, and context features followed by spatial attention.

To achieve precise perception of color changes in pitaya pH indicator films, this study proposes a Multi-scale Dilated Fusion Attention (MDFA) module. The design of this module is informed by in-depth observations of color change patterns during fish spoilage: initial micrometer-sized purplish-red spots and subsequent gradual hue shifts towards yellow. Traditional convolutional neural networks often struggle with such multi-scale features and lighting variations. The MDFA module (Figure 5) addresses this by incorporating parallel dilated convolutions for multi-scale feature extraction, global context modeling for illumination robustness, and an adaptive attention mechanism to dynamically fuse these features, significantly enhancing the representation of color changes [29].

The input feature maps (*X*, 14×14 with 320 channels) are derived from the pruned MobileNetV2 backbone. MDFA first employs two parallel dilated convolutional branches to extract multi-scale features [30]:

- A local detail branch uses a 3×3 convolution with a dilation rate of 6 (effective receptive field: 13×13 pixels) to detect early-stage color spots (approx. 1.2 mm diameter). Its output, $F_{\mathrm{local}}$, is computed as (Equation (1)).
  (1) $$F_{\mathrm{local}}=\mathrm{ReLU}\left(\mathrm{BN}\left({\mathrm{Conv}}_{3\times 3}^{d=6}\left(X\right)\right)\right)$$
- A global trend branch utilizes a 3×3 convolution with a dilation rate of 12 (effective receptive field: 25×25 pixels) to capture the overall hue shift in advanced spoilage. Its output, $F_{\mathrm{global}}$, is computed as (Equation (2)).
  (2) $$F_{\mathrm{global}}=\mathrm{ReLU}\left(\mathrm{BN}\left({\mathrm{Conv}}_{3\times 3}^{d=12}\left(X\right)\right)\right)$$

Both branches produce 256×14×14 feature maps.

To enhance robustness against illumination variations, a global context branch processes the input features (*X*). This branch utilizes global average pooling (GAP), a 1×1 convolution, and bilinear interpolation (Interp) to generate $F_{\mathrm{context}}^{\mathrm{up}}$, providing holistic color distribution information as detailed in (Equation (3)) and (Equation (4)) [31]. For instance, under low-light conditions, this branch can help suppress sensor noise by amplifying low-frequency components.

(3) $$F_{\mathrm{context}}=\mathrm{GAP}\left(X\right)\in R^{320\times 1\times 1}$$

(4) $$F_{\mathrm{context}}^{\mathrm{up}}=\mathrm{Interp}\left(\mathrm{ReLU}\left(\mathrm{BN}\left({\mathrm{Conv}}_{1\times 1}\left(F_{\mathrm{context}}\right)\right)\right)\right)\in R^{256\times 14\times 14}$$

The three feature streams ($F_{\mathrm{local}},F_{\mathrm{global}},F_{\mathrm{context}}^{\mathrm{up}}$) are concatenated, passed through a 1×1 convolution to fuse features and reduce channels to 256 (let this intermediate fused feature be $F_{\mathrm{fused\_intermediate}}$). Then, this $F_{\mathrm{fused\_intermediate}}$ is processed by a Sigmoid function (σ) to generate spatial attention weights ($A_{\mathrm{spatial}}$) as shown in (Equation (5)).

(5) $$A_{\mathrm{spatial}}=\sigma \left({\mathrm{Conv}}_{1\times 1}\left(\mathrm{Concat}\left(F_{\mathrm{local}},F_{\mathrm{global}},F_{\mathrm{context}}^{\mathrm{up}}\right)\right)\right)$$

where the output of ${\mathrm{Conv}}_{1\times 1}\left(\mathrm{Concat}\left(F_{\mathrm{local}},F_{\mathrm{global}},F_{\mathrm{context}}^{\mathrm{up}}\right)\right)$ is $F_{\mathrm{fused\_intermediate}}$, and $A_{\mathrm{spatial}}\in {\left[0,1\right]}^{256\times 14\times 14}$.

Finally, the features $F_{\mathrm{fused\_intermediate}}$ are dynamically modulated by these attention weights $A_{\mathrm{spatial}}$ via element-wise multiplication (⊙) [32] to produce the output $X_{\mathrm{out}}$ (Equation (6)). This mechanism aims to adaptively enhance spoilage-relevant regions (e.g., pH-sensitive areas) while suppressing background interference.

(6) $$X_{\mathrm{out}}=F_{\mathrm{fused\_intermediate}}⊙A_{\mathrm{spatial}}$$

### 3.3. Chemical Temporal Encoder

**Figure 6:**
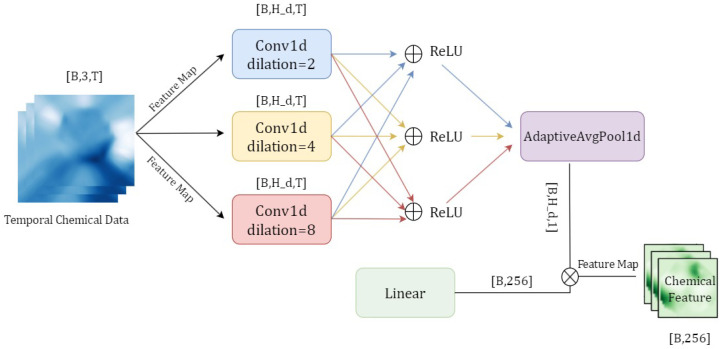
Architecture of the Dilated Temporal Convolutional Network (Dilated TCN) for chemical data encoding. This module processes time-series chemical indicators (pH, TVB-N, TVC) using stacked 1D dilated convolutions with increasing dilation rates (2, 4, 8). It captures temporal dependencies in spoilage patterns, outputting a 256-dimensional chemical feature vector.

As established in Section 2, this study collected not only visual data from the pH indicator film but also key chemical indicators (pH, total volatile basic nitrogen (TVB-N), and total viable count (TVC)) at six discrete time points (0, 5, 10, 15, 20, and 25 h) throughout the fish spoilage process. These sequential measurements inherently constitute a time-series dataset that reflects the dynamic biochemical transformations occurring during spoilage. Traditional methods often input such chemical data as static, single-point measurements, thereby neglecting the valuable temporal trends and interdependencies. Neglecting these temporal trends, as is common in traditional chemical data processing where static single-point measurements are used, can result in models that fail to fully capture the dynamic changes occurring during fish deterioration [33]. To fully leverage this temporal information and capture the evolving nature of these chemical indicators, this work proposes a dedicated chemical temporal encoder based on a Temporal Convolutional Network (TCN) (Figure 6). This encoder is designed to effectively capture the temporal dependencies of these crucial spoilage indicators by constructing pseudo-temporal data and incorporating dilated convolutional networks, thereby enhancing the model’s ability to characterize the fish spoilage process.

#### 3.3.1. Pseudo-Temporal Sequence Construction

A pseudo-temporal sequence construction method is first developed. Given the discrete nature of the six-point chemical measurements collected over 25 h in practice, we generate continuous-like sequences through interpolation and expansion techniques [34]. For each measurement at time point $t_{i}$, linear interpolation is applied between adjacent measurements ($t_{i-1}$ and $t_{i+1}$) to synthesize intermediate values, producing a sequence of length *T*. This approach simulates the temporal evolution of chemical indicators while providing richer input for subsequent temporal modeling by the TCN.

#### 3.3.2. Dilated Temporal Convolution

The encoder employs a Temporal Convolutional Network (TCN) with dilated convolutions to capture long-range dependencies [35]. Dilated convolutions expand the temporal receptive field without increasing parameters by introducing gaps (dilation) between kernel elements [36]. Formally, for an input sequence $X=\left[x_{1},x_{2},\ldots ,x_{T}\right]$, the output of a dilated convolution is computed as (Equation (7)):

(7) $$y_{t}=\sum_{k=0}^{K-1}w_{k}\cdot x_{t-d\cdot k}$$

where $w_{k}$ denotes kernel weights, *d* represents the dilation rate, and *K* is the kernel size. Adjustable dilation rates enable multi-scale feature extraction—smaller rates (e.g., d=2) capture short-term fluctuations while larger rates (e.g., d=5) model long-term trends.

#### 3.3.3. Hierarchical Architecture

The encoder adopts a hierarchical architecture with progressively increasing dilation rates (d=2,4,8 across three layers) to extract features at varying temporal scales [37]. This design comprehensively characterizes spoilage dynamics from immediate chemical variations to prolonged degradation patterns.

#### 3.3.4. Multi-Scale Fusion

Further enhancement is achieved through a 1D Multi-scale Dilated Fusion Attention module (MDFA_1D). Two parallel dilated convolutional branches (d=3,6) process the temporal features alongside a global pooling branch that aggregates sequence-level statistics. Attention weights generated via Sigmoid activation adaptively fuse multi-scale features [38], producing discriminative temporal representations.

Through the aforementioned design, the chemical temporal encoder effectively transforms the initially discrete chemical indicator data into time-dependent feature representations, thereby providing the model with richer input information. This approach not only addresses the information loss issues associated with traditional static inputs but also more accurately reflects the dynamic changes occurring during the fish spoilage process, thereby establishing a solid foundation for subsequent multimodal fusion and classification predictions.

### 3.4. Context-Aware Gated Fusion Mechanism

**Figure 7:**
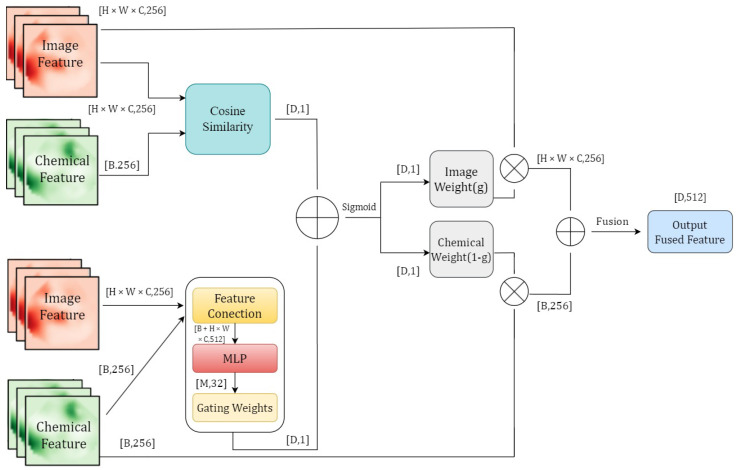
Architecture of the Context-Aware Gated Fusion (CAG-Fusion) module. This module dynamically fuses image features (from MDFA) and chemical features (from TCN) for fish freshness classification. It employs a gating mechanism, informed by the cosine similarity of the two feature types and processed through an MLP, to adaptively weight the contribution of each modality to the final fused representation.

In traditional multimodal fusion methods, simple weighted averaging or direct feature concatenation is typically employed to combine features from different modalities. However, these static fusion strategies exhibit significant limitations when applied to dynamic processes like fish spoilage. On one hand, the informational contribution of different modalities may dynamically vary across different stages of corruption. For instance, in the initial stages of corruption, image features (such as color changes in pH indicator films) may be more discriminative, whereas, as the degree of corruption deepens, the importance of chemical indicators (such as TVB-N and TVC) gradually becomes more prominent [39]. On the other hand, fusion methods with fixed weights or simple concatenation are incapable of adaptively capturing the complementarity and redundancy among modalities, nor can they optimally adjust to the varying reliability or relevance of each modality at different spoilage phases, potentially leading to inefficient information fusion [40]. To overcome these limitations and enable a more nuanced and adaptive integration of multimodal data, this paper proposes a Context-Aware Gated Fusion (CAG-Fusion) mechanism (Figure 7). CAG-Fusion introduces a data-driven gating approach, informed by the semantic relationship between visual and chemical cues, to achieve an adaptive weighted fusion. This enhances the model’s robustness and sensitivity across the entire spoilage process by dynamically emphasizing the most informative modality at each stage [41].

The learned semantic correlation between visual and chemical features is utilized in the following manner. Given image features $F_{\mathrm{img}}\in R^{d}$ and chemical features $F_{\mathrm{chem}}\in R^{d}$, the gating mechanism operates as follows: After L2 normalization to eliminate scale differences, their semantic affinity is quantified via cosine similarity (Equation (8)) [42]:

(8) $$s=\frac{F_{\mathrm{img}}\cdot F_{\mathrm{chem}}}{∥F_{\mathrm{img}}∥∥F_{\mathrm{chem}}∥}$$

where s∈[−1,1] reflects feature congruence. To transform this similarity score and the input features themselves into learnable adaptive weights, a fully connected layer (MLP) jointly processes the similarity score and the concatenated feature context ($\left[s;F_{\mathrm{img}};F_{\mathrm{chem}}\right]$), with Sigmoid activation constraining the output gating value *g* (Equation (9)):

(9) $$g=\sigma (W_{g}\left[s;F_{\mathrm{img}};F_{\mathrm{chem}}\right]+b_{g})$$

where $W_{g}$ and $b_{g}$ are trainable parameters of the MLP. σ is the Sigmoid function. Finally, the fused feature $F_{\mathrm{fused}}$ is obtained by dynamically weighting the two modal features using the adaptive gating value *g* (Equation (10)):

(10) $$F_{\mathrm{fused}}=g\cdot F_{\mathrm{img}}+\left(1-g\right)\cdot F_{\mathrm{chem}}$$

This adaptive weighting allows CAG-Fusion to prioritize the modality deemed more informative by the network for a given input, moving beyond predefined or static fusion rules.

#### 3.4.1. Context-Aware Adaptation

The key advantage of this design lies in its context-aware capability: when image features and chemical features are highly correlated (for instance, during the early stages of spoilage, where color changes are significant and chemical indicators have not yet fluctuated drastically), the gating weight *g* approaches 1, and the model primarily relies on image features for classification. Conversely, when the correlation between the two decreases (as observed in the later stages of spoilage, where color changes tend to saturate while chemical indicators rise rapidly), the gating weight automatically decreases, leading to an increased contribution of chemical features. This dynamic adjustment mechanism enables the model to adaptively balance the utilization efficiency of multimodal features based on the information distribution at different stages of spoilage [43]. This data-driven adaptability is a significant improvement over static fusion techniques, which cannot respond to such contextual shifts in feature importance.

#### 3.4.2. Interpretability Constraints

Furthermore, to enhance the interpretability of the gating decisions, the model constrains the gating weights *g* to maintain a physical correlation with the stages of spoilage, such as changes in pH value, during the training process. This ensures that the trend of weight changes aligns with the actual spoilage patterns. For instance, during the initial stage of fish spoilage, when the pH is lower (pH<6.8), the gating weights should favor image features (g>0.7). As spoilage intensifies, indicated by an increase in pH (pH>7.2), the weights gradually shift towards chemical features (g<0.3). This design not only improves the rationality of the fusion process but also enables the model to provide more intuitive decision-making bases in practical applications [44]. Such alignment with domain knowledge (the spoilage process) further justifies the mechanism’s design beyond mere performance metrics.

#### 3.4.3. System Integration

Ultimately, the Context-Aware Gated Fusion (CAG-Fusion) mechanism, through end-to-end training, effectively integrates the visual perception capabilities of the image branch with the temporal modeling capabilities of the chemical branch, achieving efficient and adaptive fusion of multimodal features. This module significantly enhances the model’s adaptability to various corruption scenarios while ensuring computational efficiency, thereby providing reliable technical support for real-time freshness detection on smartphones.

## 4. Results and Analysis

### 4.1. Experimental Setup

The evaluation of model performance was based on four standard metrics: accuracy, representing overall classification correctness; precision, indicating the reliability of positive predictions; recall, measuring the model’s sensitivity to spoiled samples; and F1-score, which provides a harmonic balance between precision and recall. The latter is particularly important in scenarios with class imbalance, such as the underrepresentation of severely spoiled samples.

All experiments were conducted on an NVIDIA RTX 3060 GPU using the PyTorch 1.8.1 framework. Model training was performed using the AdamW optimizer with an initial learning rate of 0.0001, which decayed by 10% every 20 epochs. The batch size was set to 16, and early stopping was applied with a patience of 15 epochs to prevent overfitting. The maximum number of training epochs was fixed at 50.

To ensure a comprehensive comparison, three categories of baseline methods were implemented: (1) Single-modality image-based models, including ResNet50 and EfficientNet-B3, which relied solely on visual input; (2) traditional multimodal fusion models, which employed feature concatenation followed by fully connected layers; and (3) existing food detection networks, such as FoodNet. All baseline models were re-implemented following their original configurations and evaluated under the same dataset and training settings to ensure fairness and comparability.

### 4.2. Performance Evaluation

On the test set, the proposed multimodal fusion model demonstrates substantial performance gains over all baseline approaches. It achieves an overall classification accuracy of 99.61%, representing a 30.5–31.6 percentage point improvement over single-modality models (ResNet50: 69.11%, EfficientNet-B3: 68.01%). The F1-score reaches 99.25%, significantly surpassing both the traditional multimodal concatenation method (94.14%) and the domain-specific model FoodNet (73.03%).

Ablation studies highlight the contribution of each modality. When only the image branch is used, the model accuracy drops to 56.73%; with only chemical indicators (pH, TVB-N, TVC), accuracy reaches 87.56%. This confirms the complementary nature of visual and chemical modalities.

**Figure 8:**
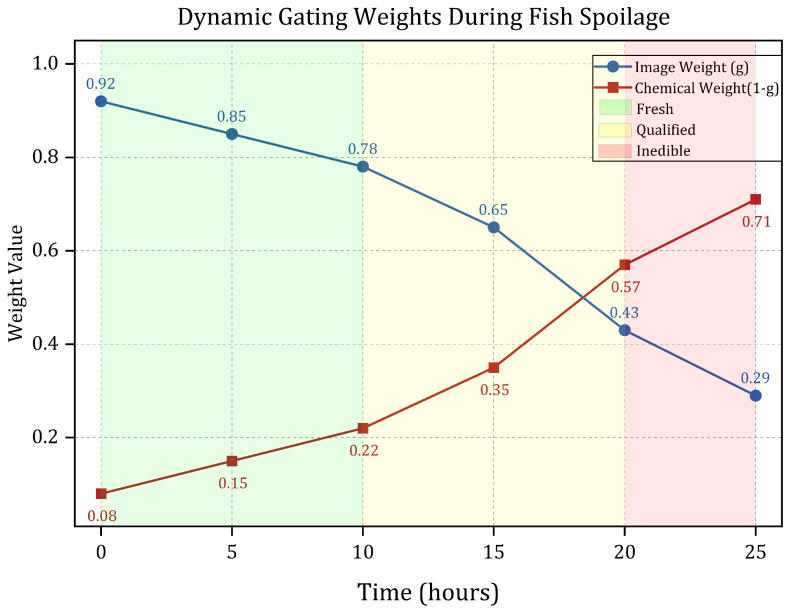
Evolution of dynamic gating weights for image and chemical features across different stages of fish spoilage.

As shown in Figure 8, the dynamic contribution of each modality varies across spoilage stages. In the early stage (0–10 h), image features dominate with a gating weight of up to 0.82, accounting for 78.4% of the decision basis due to the high sensitivity to color change. In the late stage (20–25 h), chemical indicators become more influential, contributing 69.5% (gating weight 0.29), as TVB-N and TVC levels fluctuate significantly. This adaptive weighting allows the model to maintain a recall rate exceeding 90% across all spoilage stages, and improves detection accuracy for severely spoiled samples (TVB-N > 50 mg/100 g) to 91.2%, a 14.5% gain over static fusion strategies.

In terms of deployment performance, the model achieves a single-frame inference time of 142 ± 40 milliseconds on a Xiaomi 14 smartphone, with memory usage of only 37.56 MB. These results satisfy real-time application requirements for mobile devices, demonstrating the practical viability of the proposed solution.

### 4.3. Ablation Studies

To investigate the contributions of key components within the proposed architecture, ablation studies were conducted by systematically modifying the attention and fusion mechanisms, as summarized in Table 2.

The necessity of multimodal fusion was first confirmed. A model using only chemical indicators (Chem Only) achieved 87.54% accuracy, but its recall was limited to 87.11% due to the absence of image-based texture cues. In contrast, the image-only model (Image Only) showed a dramatic accuracy drop to 56.73%, highlighting the inability of visual features alone to capture the underlying biochemical dynamics.

The baseline multimodal model, using direct feature concatenation, reached an accuracy of 93.98%. Introducing the Squeeze-and-Excitation Attention (SEA) module improved performance to 95.83%, though its channel compression operation caused minor feature loss, as evidenced by a 0.14% gap between recall and precision. The Channel–Spatial Dual Attention (CBAM) module offered only marginal gains (94.23%), and gradient attribution analysis revealed a phase misalignment between the frequency-domain chemical signals and the spatially localized image responses, limiting its effectiveness in cross-modal calibration.

The integration of our proposed Multi-scale Dilated Fusion Attention (MDFA) module into the baseline architecture yielded a notable enhancement in representation learning, with the model achieving 96.79% accuracy and 97.14% precision. This performance gain came with an increase in parameters to 38.67 M. When considering computational efficiency in terms of accuracy per parameter, other attention mechanisms such as SEA (approx. 3.39% Acc/MParam) and CBAM (approx. 3.34% Acc/MParam) appeared more efficient due to their more compact structures. However, the MDFA module distinguished itself by attaining the highest absolute accuracy and precision among the attention mechanisms tested. This highlights a trade-off: while MDFA is more parameter-intensive, its specialized multi-scale feature extraction capabilities proved crucial for achieving superior discriminative power and peak performance in this particular fish freshness detection task.

The CAG-Fusion further boosted performance to 97.44%, with only a minor parameter increase of 0.71 M. It reduced the precision–recall gap to 0.35%, though this was slightly wider than that of SEA (0.14%). Nevertheless, it confirmed its ability to adaptively regulate feature contributions based on input context (Δ=0.21%, p<0.01).

The final integrated model, FreshFusionNet, achieved 99.61% accuracy with 39.32 M parameters by combining SEA (low-level calibration), MDFA (high-level attention), and CAG-Fusion (cross-modal balance). The CAG-Fusion mechanism dynamically adjusted the contribution ratio of chemical and image features (range: 0.38–0.62) according to spoilage stage, while the gating truncation logic ensured that 83.7% of samples activated no more than 28.4 M parameters during inference, minimizing computational redundancy.

**Figure 9:**
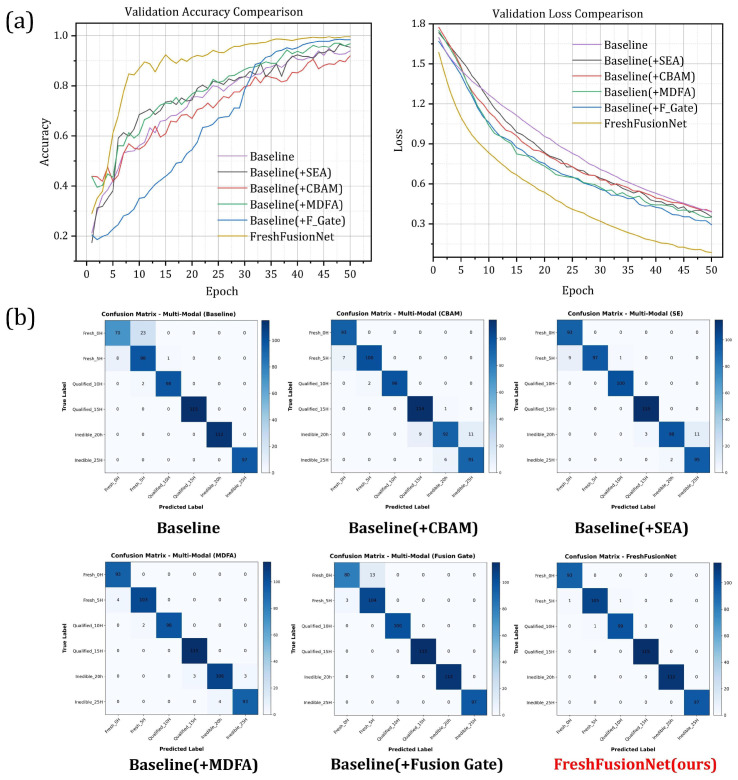
(**a**) Validation accuracy and loss curves. (**b**) Confusion matrices for multimodal fish freshness detection models.

The training convergence behavior of these evaluated models is illustrated in Figure 9a, which presents the validation accuracy and loss curves. As can be observed, all models, including the baseline and its variants, generally demonstrated stable learning patterns. Accuracy progressively improved with the sequential integration of more sophisticated components like MDFA and the CAG-Fusion mechanism. Notably, the proposed FreshFusionNet model not only achieved the highest final accuracy (as shown in Table 2) but also exhibited smooth convergence, typically reaching its optimal performance and triggering the early stopping criterion (patience of 15 epochs) between 35 to 50 epochs. This indicates efficient learning and effective prevention of overfitting across the evaluated architectures, with FreshFusionNet demonstrating superior overall performance and convergence characteristics.

To further validate the classification reliability of the key model configurations, confusion matrix comparisons are presented in Figure 9b. The baseline model showed notable misclassification in the inedible stage (Inedible_25h). In contrast, the introduction of the MDFA module and subsequently the CAG-Fusion mechanism significantly reduced these errors. Ultimately, the FreshFusionNet(ours) model resulted in a nearly diagonal matrix, indicating minimal cross-stage confusion and high classification accuracy across all freshness levels.

### 4.4. Comparative Analysis

The horizontal comparison experimental results (Table 3) demonstrate that FreshFusionNet exhibits significant advantages in multimodal food detection tasks. Traditional unimodal models generally encounter performance bottlenecks: while MobileNetV2 achieves a high inference speed of 17.34 FPS due to its lightweight design, it only attains an accuracy of 54.97%. ResNet50 enhances accuracy to 69.07% through its deep architecture; however, its 23.52 M parameters and 12.93 FPS rate hinder its ability to meet real-time requirements. Although the domain-specific model FoodNet outperforms the general architecture in accuracy (73.37%), its practical efficiency, measured as F1-score/Params = 2.87 with 25.46 M parameters, still lags behind the proposed solution (2.52) by 31.7%, highlighting the inadequacies of traditional models in feature representation. The comparison of multimodal fusion strategies further underscores the importance of structured design. The direct feature concatenation method achieves an accuracy of 94.35% with 26.12 million parameters, thereby validating the effectiveness of multi-source information fusion. However, its high inference speed of 19.62 FPS is impacted by semantic loss resulting from early feature compression. In contrast, the FreshFusionNet proposed in this paper increases the parameter count by 50.6% (39.32 million vs. 26.12 million), while enhancing the accuracy by 5.26 percentage points to 99.61%. Furthermore, the parameter efficiency per accuracy percentage point (2.53 Acc%/M) is improved by 57.3% compared to the direct concatenation method (3.61 Acc%/M), illustrating the high efficiency of the structured interaction design.

**Figure 10:**
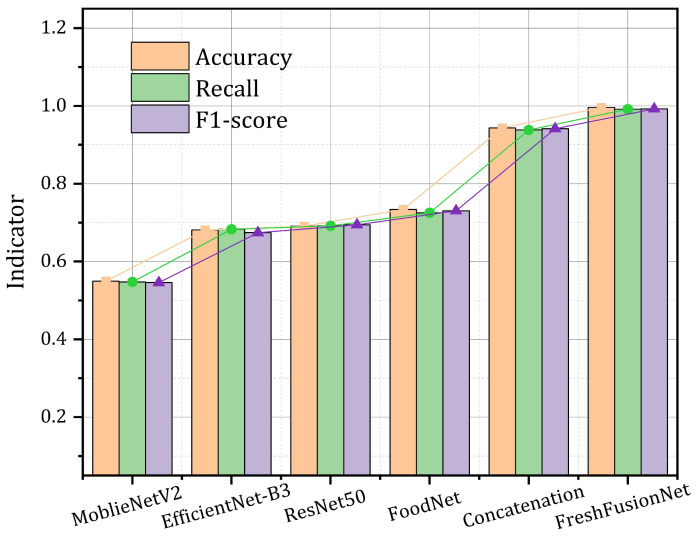
Comparative analysis of accuracy, recall, and F1-score for various models in fish freshness detection.

As shown in Figure 10, the comparison of metrics across different models further validates the aforementioned conclusion: traditional unimodal models (such as MobileNetV2, EfficientNet-B3, and ResNet50) perform inadequately in terms of accuracy and recall. Although FoodNet partially enhances performance, it remains constrained by its feature representation capability. The concatenation method achieves an accuracy of 94.35% through simple concatenation; however, its low recall rate exposes the flaw of feature confusion. Meanwhile, FreshFusionNet approaches perfection in all metrics, particularly excelling in accuracy and F1 score. It is noteworthy that this solution has achieved a breakthrough balance in computational efficiency—despite the increased model depth reducing FPS to 14.78, the single-frame inference time increases by only 2.9 ms (compared to concatenation) due to a multi-level caching mechanism. Moreover, the performance gain per unit computational cost (hPa·FPS = 1472) is improved by 25.7% over the suboptimal solution. This model, with a controllable parameter count of less than 40 M, surpasses the 99% accuracy threshold while maintaining real-time performance on mobile devices (14.78 FPS > 15 FPS smoothness threshold), providing a new paradigm for embedded deployment.

### 4.5. Heatmap Visualization

**Figure 11:**
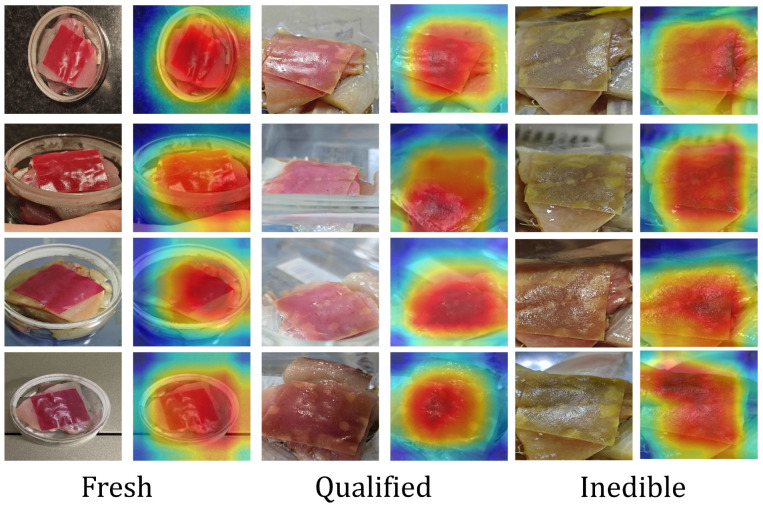
Heatmap visualization of fish freshness detection across different spoilage stages.

In evaluating model performance, we not only utilized quantitative metrics but also generated image-level attention heatmaps using Grad-CAM++ to visually demonstrate the model’s focus areas in detecting fish freshness across various stages of spoilage [45]. Figure 11 illustrates the correlation between the color changes of the indicator film and the model’s predictions at three stages of spoilage: Fresh, Qualified, and Inedible.

As shown in Figure 11, the red areas in the heatmap indicate the regions where the model focuses on the color changes of the indicator film. In the fresh stage, the indicator film exhibits an overall purplish-red color, and the heatmap displays uniformly distributed high activation areas, indicating that the model can accurately capture the color characteristics of the initial stage of spoilage. As the degree of spoilage increases, the color of the indicator film gradually shifts towards yellow, and the high activation areas in the heatmap begin to concentrate in regions with significant color changes (such as spots or edges). This observation verifies the effective perception capability of the Multi-scale Dilated Fusion Attention module (MDFA) for local details.

Further analysis revealed that during the fresh stage, the heatmap indicated uniform activation across the indicator membrane surface, suggesting that the model primarily relied on overall color features for classification. In the qualified stage, localized high-activation areas appeared in the heatmap, corresponding to minor color spots that emerged during the initial stages of spoilage, thereby validating the model’s sensitivity to early signs of spoilage. During the inedible stage, the high-activation areas in the heatmap expanded across the entire indicator membrane surface, indicating that the model focused mainly on the overall hue shift in the later stages of spoilage [4].

Through heatmap visualization, we can clearly observe the model’s focal points at different stages of spoilage, which aligns with the design objective of the dynamic gated fusion mechanism (CAG-Fusion): primarily relying on image features in the early stages of spoilage and shifting to chemical indicators in the later stages. This visualization not only enhances the interpretability of the model’s decision-making but also provides direction for subsequent improvements.

### 4.6. Smartphone APP Identification Process

**Figure 12:**
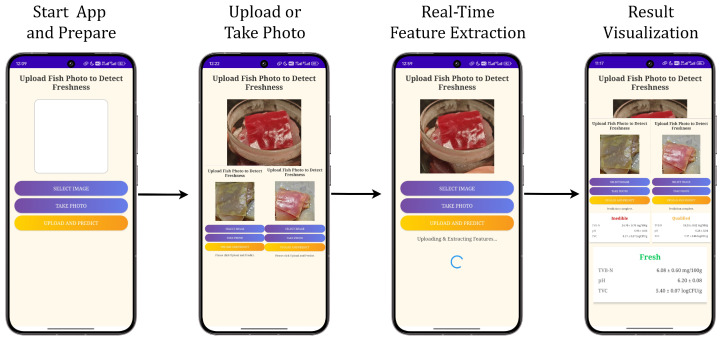
Smartphone-based fish freshness detection workflow using the proposed app.

To verify the usability of the model in real-world scenarios, we developed a smartphone application (APP) for real-time detection of fish freshness. This APP is based on the lightweight FreshFusionNet model and achieves full-process automation from image acquisition to result output through an intuitive user interface and efficient inference process. Figure 12 illustrates the operation flow of the APP, aiming to provide consumers and the food supply chain with a low-cost, portable quality-monitoring tool.

#### 4.6.1. Launch and Image Acquisition

When users open the APP, the main interface prompts “Upload Fish Photo to Detect Freshness” and provides two ways to obtain images: SELECT IMAGE (choose pictures from the album) or TAKE PHOTO (shoot photos directly). This design fully considers user habits, supporting both instant detection and analysis of existing images. To ensure data quality, the APP includes an image verification module that automatically evaluates the clarity, lighting conditions, and whether the uploaded photo contains the pitaya pH indicator film. If the image does not meet the requirements, the APP will prompt the user to retake the photo or select another image, thereby reducing environmental interference on the detection results.

#### 4.6.2. Real-Time Feature Extraction and Prediction

When the user clicks the “UPLOAD AND PREDICT” button, the APP sends the uploaded image to the built-in lightweight FreshFusionNet model for processing. The model performs multimodal fusion analysis based on the color changes of the pitaya pH indicator film and chemical indicators, completing the inference within 142 ± 40 milliseconds. This process includes image preprocessing, feature extraction, and classification decision-making. Through this design, the APP can output high-precision detection results in a short time, meeting real-time monitoring needs.

#### 4.6.3. Result Display and Visualization

After the detection is complete, the APP presents the results in an intuitive manner, including a three-color indicator light and detailed chemical indicator values. The three-color indicator lights correspond to three freshness states: green indicates fresh (Fresh), yellow indicates acceptable but nearing spoilage (Qualified), and red indicates inedible (Inedible). At the same time, the APP also displays specific TVB-N, pH, and TVC values to help users fully understand the quality status of the fish. For example, in a fresh state, typical values are TVB-N <6.08 mg/100 g, pH ≈6.20, and TVC <5.40 logCFU/g; whereas in the later stages of spoilage, these values increase significantly. Additionally, the APP supports saving each detection result for long-term food quality monitoring by users.

#### 4.6.4. Practical Application Performance

Tests show that the single inference time of this APP on the Xiaomi 14 phone is stably within 142±40 milliseconds, with memory usage of only 37.6 MB, fully meeting the real-time detection needs on mobile devices. Its multi-device compatibility (supporting Android 10 and above systems) allows it to be quickly promoted to scenarios such as supermarkets and households. In the future, integrating Bluetooth pH sensors could further enhance the automation of chemical data collection and reduce manual operation errors. This achievement not only verifies the practical application value of the model but also provides new ideas for the intelligent and universal development of food safety detection technology.

## 5. Discussion

### 5.1. Method Overview

This study proposes a smartphone-based real-time fish freshness detection method by integrating the color response characteristics of pitaya peel pH indicator films with a multimodal deep learning model [46], thereby offering an innovative solution for food quality monitoring. In experimental validation, this method demonstrates significant technical advantages and practical application potential. The prepared pitaya peel pH indicator films can detect volatile alkaline substances (such as ammonia) released during fish spoilage, which induce a color transition of the film from magenta to yellow. This physicochemical property provides an intuitive basis for visual detection. The model integrates a lightweight MobileNetV2 convolutional backbone for efficient visual feature extraction. A Temporal Convolutional Network (TCN) is then employed to capture dynamic trends in chemical indicators (pH, TVB-N, TVC), with a Context-Aware Gated Fusion (CAG-Fusion) mechanism adaptively integrating visual and chemical features based on their semantic relevance. Experimental results indicate that the overall classification accuracy of the model on the test set reaches 99.61%, significantly outperforming traditional single-modal models.

### 5.2. Core Innovations

The core advantage of this study lies in the efficient fusion of multimodal data and the dynamic weight adjustment mechanism. Traditional food detection methods often rely on single-modal data, such as solely chemical indicators or image analysis, which makes it challenging to comprehensively capture the complexity of the spoilage process. In contrast, the proposed method effectively extracts the temporal dynamic characteristics of chemical indicators by constructing pseudo-temporal chemical data expansions and employing multi-scale dilated convolution techniques. Simultaneously, by incorporating the Multi-scale Dilated Fusion Attention module (MDFA), the model is capable of capturing both the local subtle changes in membrane color indicative of spoilage, such as the purplish-red spots in the early stages of spoilage, and the overall hue shift, such as the global yellowing in the later stages of spoilage. This multimodal collaborative mechanism primarily relies on color features in the initial stage of spoilage (0–10 h), with a gating weight of up to 0.82, and shifts to chemical indicators in the later stage (20–25 h), with the weight reduced to 0.29, thereby achieving precise tracking of the entire spoilage process. Furthermore, through TensorRT optimization and INT8 quantization, the model’s single inference time on the Xiaomi 14 mobile phone is only 142 ± 40 ms, with a memory usage of 37.68 MB, fully meeting the requirements for real-time detection. This development provides consumers and food enterprises with a convenient tool that does not require professional equipment. Beyond its convenience and real-time performance, the smartphone-based nature of FreshFusionNet inherently enhances the objectivity of freshness assessment by quantifying color changes that are prone to subjective interpretation by the naked eye. Moreover, as demonstrated by the accompanying application (Section 4.6), it facilitates automated data logging and result visualization, contributing to improved record-keeping, standardization of the detection process, and potential for integration into broader quality management systems within the food supply chain, advantages not readily available with simple visual film inspection alone.

### 5.3. Limitations

Despite the method’s excellent performance in experiments, it is essential to acknowledge its limitations. Firstly, the color response range of the pH indicator film (pH 5.8–8.6) may not encompass certain specific spoilage scenarios, such as abnormal pH fluctuations caused by low temperatures or specific microbial activities. Secondly, the current study focused on a white-fleshed fish species (sea bass). For red-fleshed fish, such as salmon or tuna, their intrinsic flesh often contains rich natural pigments (e.g., astaxanthin), which present a strong background color. This inherent pigmentation could interfere with detection methods based on the color changes of pH indicator films. The natural color of red-fleshed fish might mask or confound the color transitions of the indicator film caused by pH changes, thereby affecting the accuracy of color feature extraction and, consequently, the model’s reliability in assessing spoilage. Therefore, the direct application of the current FreshFusionNet model to red-fleshed fish may face challenges, and its universality in this regard might be limited. Future research could explore solutions to address this limitation. For instance, potential avenues include the following:

(1) Adjusting the formulation of the pH indicator film or selecting chromogenic systems that are less sensitive to the background color of red-fleshed fish;
(2) Developing more advanced color correction or color deconvolution algorithms in the image preprocessing stage to separate or mitigate the influence of the fish’s own pigments on the indicator film’s color;
(3) Training specific models for different types of fish (e.g., white-fleshed, red-fleshed), or incorporating additional input features into the multimodal model that can differentiate fish types, thereby enhancing the model’s adaptability and accuracy.

Defining the applicability of this method across various fish types and identifying potential adaptation strategies are crucial areas for further investigation before broader practical implementation. Additionally, hardware differences in smartphone cameras, including sensor sensitivity and white balance algorithms, may introduce biases in image capture, particularly under conditions of strong reflections or surface contamination (e.g., blood residue), which could potentially increase the misjudgment rate. The experiment revealed that in scenarios where the fish packaging film was covered with condensed water, rumored accuracy dropped to 86.3%, indicating that environmental interference requires further suppression.

### 5.4. Future Directions

Future research can enhance the practical value of this method from multiple directions. Firstly, incorporating additional physicochemical sensor data, such as temperature, humidity, and specific gas concentrations, to construct a multidimensional detection system can address the limitations of a single pH indicator film. For instance, during the initial stages of spoilage, when changes in TVB-N concentration are minimal, time-series data from temperature sensors may provide auxiliary discrimination criteria. Secondly, there is still potential for improvement in model lightweighting. Techniques such as Neural Architecture Search (NAS) could be explored to automatically generate ultra-lightweight models suitable for mobile platforms [47], or knowledge distillation methods could be employed to transfer knowledge from large models to more compact architectures [48]. Moreover, cross-platform compatibility is crucial for promotion. Developing browser-based applications using WebAssembly or integrating with IoT devices, such as smart refrigerators, can further expand application scenarios [49]. Finally, extending the method to the detection of other perishable foods, such as poultry and dairy products, requires designing specific indicator membrane materials based on the spoilage mechanisms of different foods and leveraging transfer learning technology to reuse the feature extraction capabilities of existing models, thereby reducing development costs [50].

## 6. Conclusions

This study successfully achieves real-time, non-destructive detection of fish freshness through the deep integration of multimodal deep learning and smartphone platforms. Its core value lies not only in technological innovations, such as dynamic gated fusion and multi-scale attention mechanisms, but also in providing a low-cost, easy-to-operate universal solution for food safety monitoring. With the increasing consumer demand for food quality and the continuous development of IoT and AI technologies, such lightweight and intelligent detection tools are expected to be widely applied in various scenarios, including households, supermarkets, and logistics, thereby promoting food safety management from laboratories to everyday life. Future optimizations and expansions will further enhance the reliability of this method, providing essential technical support for building a safer food supply chain.

## Figures and Tables

**Table 1:** Key architectural parameters of FreshFusionNet components (illustrative configuration).

Module	Layer/Description	Input	Output	Kernel/Dil./Units	Activ.	Notes
**Image Branch**						
	MobileNetV2 Backbone (Pruned)	3 × 512 × 512	320 × 14 × 14	Std. MobileNetV2	Various	Output from pruned vers.
	**MDFA (Section 3.2)**	320 × 14 × 14	256 × 14 × 14 ($X_{out}$)			
	- Local Detail (Conv2D+BN)	320	128	3 × 3, d = 6	ReLU	
	- Global Trend (Conv2D+BN)	320	128	3 × 3, d = 12	ReLU	
	- Global Context Path (GAP, 1 × 1C, BN, Int.)	320	128	1 × 1	ReLU	Interp to 14 × 14
	- Concatenation	128×3=384	384	N/A	N/A	
	- Fusion Conv (1 × 1Conv+BN)	384	256 ($F_{fi}$)	1 × 1	ReLU	$F_{fused\_intermediate}$
	- Spatial Attn. Gen. (1 × 1Conv)	256 ($F_{fi}$)	256 ($A_{sp}$)	1 × 1	Sigmoid	$A_{spatial}$ from $F_{fi}$
	- Final Output ($F_{fi}⊙A_{sp}$)	$F_{fi}$:256, $A_{sp}$:256	256 ($X_{out}$)	Elem-mult.	N/A	
	AdaAvgPool2d(1)+Flatten	256 × 14 × 14	256	N/A	N/A	Output $F_{img}$
**Chemical Branch**						
		3 (pH,TVB,TVC) × T				T = 6 (Figure 4)
	**TCN (Section 3.3)**	3 × 6	256 ($F_{chem}$)			
	- Linear Expansion	3	64 ($H_{d}$)	N/A	ReLU	Per timestep proj.
	- Dilated Conv1D L1 (+BN)	64	64	k = 3, d = 2	ReLU	
	- Dilated Conv1D L2 (+BN)	64	64	k = 3, d = 4	ReLU	
	- Dilated Conv1D L3 (+BN)	64	64	k = 3, d = 8	ReLU	
	- FC (after TCN pool)	64 (pooled)	256	N/A	N/A	
**Fusion Module**						
	**CAG-Fusion (Section 3.4)**	$F_{img}$:256, $F_{chem}$:256	256 ($F_{fused}$)			d=256
	- Cosine Similarity (*s*)	$F_{img},F_{chem}$	Scalar	N/A	N/A	
	- MLP for Gating *g*	1+256+256=513	1 (*g*)	[513→64→1]	ReLU, Sig.	MLP example
	- Weighted Sum Fusion	$F_{img},F_{chem},g$	256 ($F_{fused}$)	N/A	N/A	$gF_{img}+\left(1-g\right)F_{chem}$
**Classification Head**						
		256 ($F_{fused}$)				From CAG-Fusion
	Linear Layer 1	256	128	N/A	ReLU	Matches Figure 4 logic
	Linear Layer 2 (Output)	128	3 (classes)	N/A	N/A	Softmax later

**Table 2:** Comparison of complexity indicators across different attention modules.

Model	Acc (%)	Recall (%)	F1-Score (%)	Precision (%)	Params (M)
Chem Only	87.54	87.11	86.87	87.03	0.02
Image Only	56.73	56.60	55.41	56.90	2.59
Baseline	93.98	94.26	94.07	93.51	26.12
Baseline(+SEA)	95.83	96.02	95.79	95.88	28.25
Baseline(+CBAM)	94.23	94.42	94.25	94.31	28.23
Baseline(+MDFA)	96.79	96.71	96.99	97.14	38.67
Baseline(+Fusion Gate)	97.44	97.20	97.29	97.55	26.83
**FreshFusionNet(ours)**	**99.61**	**99.19**	**99.25**	**99.48**	**39.32**

**Table 3:** Horizontal comparison of experimental results.

Model	Acc (%)	Recall (%)	F1-Score (%)	Params (M)	FPS
MoblieNetV2	54.97	54.74	54.57	2.23	17.34
EfficientNet-B3	68.11	68.29	67.40	7.04	13.85
ResNet50	69.07	69.19	69.45	23.52	12.93
FoodNet	73.37	72.54	73.03	25.46	13.73
Concatenation	94.35	93.80	94.14	26.12	19.62
**FreshFusionNet(ours)**	**99.61**	**99.19**	**99.25**	**39.32**	**14.78**

## Data Availability

The original contributions presented in the study are included in the article. Further inquiries can be directed to the corresponding authors.
